# Building Scientific Writing and Publication Capacity of New Authors from Low- and Middle-Income Countries: A Multicomponent Global Collaboration Model

**DOI:** 10.5334/gh.1508

**Published:** 2025-12-23

**Authors:** Qaiser Mukhtar, Sushama D. Acharya, Andrew E. Moran, Daniel T. Lackland, Carl Reddy, Dinesh Neupane, Elizabeth Krajan Pardo, Birgit Bolton, Patricia Richter, Michael H. Olsen

**Affiliations:** 1 Centers for Disease Control and Prevention, Global Health Center, Division of Global Health and Protection, Atlanta, Georgia, USA; 2 CDC Foundation, Noninfectious Disease Programs, Atlanta, Georgia, USA; 3 Resolve to Save Lives, Cardiovascular Health, New York, USA; 4 Columbia University, Irving Medical Center, New York, USA; 5 Medical University of South Carolina, Charleston, South Carolina, USA; 6 The Task Force for Global Health, Training Programs in Epidemiology and Public Health Interventions Network, Atlanta, Georgia, USA; 7 Department of International Health, Bloomberg School of Public Health, Johns Hopkins University, Baltimore, Maryland, USA; 8 Alexton, Inc. Lorton, Virginia, USA; 9 Department of Internal Medicine, Holbaek Hospital, Copenhagen, Denmark; 10 Department of Clinical Medicine, University of Copenhagen, Copenhagen, Denmark

**Keywords:** low- and middle-income countries, cardiovascular diseases, scientific writing and publication capacity building, mentorship

## Abstract

Peer-reviewed publications using local data are critical for understanding disease burdens, generating evidence, and shaping policies tailored to community needs. Although low- and middle-income countries (LMICs) account for 80% of cardiovascular disease (CVD) deaths, they contribute only 2.8% of CVD publications. To address this gap, the US Centers for Disease Control and Prevention (CDC) and partners launched the Emerging Authors Program for Global Cardiovascular Research (EAP), supporting early- to mid-career LMIC practitioners. EAP coordinated mentorship, provided virtual writing tools, training, technical support, and financial assistance for open access. Between 2019 and 2023, three waves engaged 33 authors from 11 countries, resulting in 31 published manuscripts in six journals, with support from 23 global mentors. The success was driven by committed mentors, motivated authors, local collaboration, accessible resources, and strong communication. Strengthening LMIC authors’ writing and publication skills is essential for advancing rigorous research and global health equity in scientific publishing.

## Background

Peer-reviewed publications are the gold standard for sharing scientific knowledge. Manuscripts from low- and middle-income countries (LMICs) offer critical insights into local health challenges, informing targeted interventions and contributing to global learning. For public health practitioners, publishing also advances careers by showcasing research, writing, and communication skills. However, practitioners in LMICs face limited opportunities to publish, especially in noncommunicable disease (NCD) research. Cardiovascular diseases (CVDs) are the leading cause of NCD deaths, responsible for 19.4 million deaths in 2021—more than all communicable and maternal conditions combined (1). LMICs carry 80% of global CVD deaths and over half of the disability-adjusted life years lost, yet account for only 2.8% of CVD publications (2,3). Addressing this imbalance requires strategies that strengthen research and publication capacity in LMICs.

To bridge this gap, the US Centers for Disease Control and Prevention (CDC) and global partners developed a workforce model through Field Epidemiology Training Programs (FETPs). These country-owned programs enhance epidemiology and program management skills through classroom learning (25%), fieldwork (75%), and mentorship among national, state, and district-level health officers. In 2018, CDC received a gift fund to introduce CVD-focused FETPs in China, Ethiopia, India, and Thailand to strengthen expertise in CVD epidemiology, data analysis, evaluation, and scientific writing. The FETP details and programmatic principles are described elsewhere (4). Building on this foundation, CDC established the Emerging Authors Program for Global Cardiovascular Research (EAP) to further enhance scientific writing and publication capacity. This article describes the EAP framework, achievements, and lessons learned.

## Methods

### EAP Program Overview

EAP is a multicomponent initiative for early- and mid-career public health professionals from LMICs working on CVDs and risk factors. Implemented between 2019 and 2023 in three waves, it was a collaboration between CDC, the Lancet Commission on Hypertension Group, Resolve to Save Lives, the World Hypertension League, CDC Foundation, and Training Programs in Epidemiology and Public Health Interventions Network (TEPHINET). The program’s goal was to mentor LMIC authors through manuscript development and publication (5).

### Recruitment and Selection

For each wave, applicants submitted abstracts of unpublished manuscripts. Program structure and eligibility by wave are outlined in Table 1. Eligible participants were early- to mid-career professionals in leadership track roles in ministries of health and other national-level government or non-governmental agencies in LMICs. They were required to have fewer than four first-authored publications, be committed to writing, revising, and completing submissions within six months. After confirming eligibility, two reviewers independently evaluated each submission. Using predefined criteria, reviewers scored the methodological rigor of the research project’s abstract, including the clarity of objectives, appropriateness of study design and analysis, and relevance to CVD prevention and control. Final selection considered methodological quality, geographic representation, and programmatic priorities to help inform national CVD strategies.

**Table 1:** The Emerging Authors Program for Global Cardiovascular Research (EAP) Structure and Accomplishments, 2019–2023.


LAUNCH, YEAR	WAVE I	WAVE II	WAVE III

	2019	2021	2022

**Key partners**	CDC, CDC Foundation, LCOHG, RTSL, WHL, TEPHINET		

**Program participants**	FETP trainees or recent graduates; Public health trainees and practitioners working on RTSL-supported cardiovascular health initiatives in LMICs		FETP current trainees or recent graduates

**Scientific writing training/tools**	In-person workshop in Atlanta, USA	Online self-paced Writing a Scientific Manuscript course	Online self-paced Writing a Scientific Manuscript course Eight-part virtual series on scientific writing and publishing

**Applicants (n)**	34	48	23

**Authors selected (n)**	24	16	11*

**Discontinued (n)**	10	4	1

**Rejected (n)**	0	1	1

**Global mentors (n)**	13	8	9

**Publications (n)**	15	11	9

**Article type (n)**	Original Research (13)Editorial (2)	Original Research (9)Editorial (2)	Original Research (8)Research brief (1)

**Journal, impact factor, 2024**	Journal of Clinical Hypertension (2.5)	Journal of Human Hypertension (3.4)International Journal of STD & AIDs (1.3)	Preventing Chronic Disease (3.9)BMC Primary Care (2.6)Annals of Ibadan Postgraduate Medicine (nr)

**Authors and countries**	Fourteen authors from five countries/regions The Americas (2) India (5) Thailand (4) Eurasia (1) Pakistan (1)	Nine authors from six countries/regions Uganda (3) Tunisia (2) Nigeria (1) West Africa (1) China (1) India (1)	Nine authors from eight countries Ethiopia (1) India (1) Nigeria (2) Pakistan (1) Sierra Leone (1) Thailand (1) Uganda (1) Vietnam (1)


CDC, US Centers for Disease Control and Prevention; LCOHG, Lancet Commission on Hypertension; RTSL, Resolve to Save Lives; WHL, World Hypertension League; TEPHINET, Training Programs in Epidemiology and Public Health Interventions Network; FETP, Field Epidemiology Training Program; LMICs, low-and middle-income countries; nr = not reported. *Two authors from Wave II joined Wave III.

### Implementation

Key program components included:

- **Mentorship:** Authors were matched with global and local mentors experienced in CVD research and scientific publishing. Mentors guided manuscript development, provided feedback, and guided the peer review process. Local mentors contributed context-specific knowledge and support.
- **Training and Tools:** CDC and partners developed evolving writing tools and resources that expanded across waves.
  - *Wave I (2019):* Authors attended a three-day in-person scientific writing workshop in Atlanta, USA. Participants requested broader access to the workshop, prompting CDC and partners to launch a free, self-paced online ‘Writing a Scientific Manuscript’ course in September 2020, available worldwide in English, Spanish, and French on the TEPHINET website (6).
  - *Wave II (2021):* COVID-19 restrictions prevented an in-person workshop, but the new self-paced course supported authors through writing, submission, and review. Initially intended for FETPs only, the course exceeded expectations with 59,000 visits from 176+ countries and 20,000 downloads by December 2024.
  - *Wave III (2022):* To address the limits of self-paced tools, CDC and partners launched an eight-part webinar series on ‘Writing and Publishing’ to allow interactivity and opportunity for authors and others to ask questions, drawing over 500 participants per session from 82 countries (7).
- **Coordination and Support:** The CDC team provided technical assistance, tracked progress, facilitated communication between mentors and authors, and ensured manuscripts met journal standards.
- **Financial Support:** While mentors and authors were unpaid, the CDC Foundation funded editorial services and article processing charges (APCs) when needed.

## Results

Across the three waves, EAP supported 51 authors from 11 countries. Fifteen withdrew due to competing demands, mainly related to COVID-19 or challenges in meeting the program deadlines. The remaining unique 35 authors (two participated in two waves and two co-authored one manuscript) received mentorship. Collectively, of the 33 manuscripts submitted, 31 were published in six peer-reviewed journals, and two were rejected (Table 1). For the two rejected manuscripts, authors were advised to pursue alternative journals and received continued program support until the authors opted not to proceed. Journal impact factors for 2024 ranged from not reported to 3.9, with Preventing Chronic Disease and the Journal of Human Hypertension having the highest impact factors among the journals represented (Table 1). Google Scholar citation counts for the 31 manuscripts ranged from 0 to 71. Nine publications had fewer than 10 citations each, 18 had 10–50, and 4 had more than 50 (Table 2). Additionally, EAP partners published four editorials (Table 2).

**Table 2:** List of The Emerging Authors Program for Global Cardiovascular Research (EAP) -supported publications, 2020–2023.


EAP AUTHOR	TITLE, LINK TO ARTICLE, CITATION METRICS*	MENTOR(S)

**Wave III**		

**Azuka S Adeke**	Assessment of Task Sharing Pilot Focused on the Control of Hypertension in Primary Health Care Facilities, Ogun and Kano States, Nigeria, 2022; A Cross-Sectional Study. Link to article (0)	Dinesh Neupane^1^

**Thang Nghia Hoang**	Assessment of Availability, Readiness, and Challenges for Scaling-Up Hypertension Management Services at Primary Healthcare Facilities, Central Highland Region, Vietnam, 2020. Link to article (10)	Chaisiri Angkurawaranon^1^

**Nadia Noreen**	Determinants of Adherence to Antihypertension Medications among Patients at a Tertiary Care Hospital in Islamabad, Pakistan, 2019. Link to article (25)	Dagmara Hering^1^

**Ashish Krishna**	Understanding the Role of Staff Nurses in Hypertension Management in Primary Care Facilities in India: A Time-Motion Study. Link to article (14)	Dagmara Hering^1^

**Ikenna Onoh**	Prevalence and Predictors of Tobacco Use Among Adolescents in Ibadan, Nigeria. Link to article (8)	Cynthia Cassel^2^

**Daniel Bekele Debela**	Effect of an Educational Intervention on Lifestyle Modification of Patients with Hypertension at Bishoftu General Hospital, Ethiopia, 2021. Link to article (16)	Angelo Scuteri^1^

**Jackline Nanono**	Service Availability and Readiness of Primary Care Health Facilities Offering Hypertension Diagnosis Services in Wakiso District, Uganda, 2019. Link to article (13)	Dinesh Neupane^1^

**Chanatip Chailek**	Availability and Price of Low-Sodium Condiments and Instant Noodles in the Bangkok Metropolitan Region. Link to article (9)	Veronica Lea^2^

**Umaru Sesay**	Evaluation of a Hypertension Surveillance System, Kenema Government Hospital, Sierra Leone, 2021. Link to article (8)	Sushama Acharya^3^

**Wave II**		

**Fortunate Ambangira**	Diabetes Mellitus Care Cascade among a Cohort of Persons Living with HIV and Hypertension in Uganda: A Retrospective Cohort Study. Link to article (3)	James Sharman^1^

**Wenrong Zhang**	Knowledge and Practices Related to Salt Consumption in China: Findings from a National Representative Cross-sectional Survey. Link to article (3)	Dinesh Neupane,^1^James Sharman^1^

**Douglas Joseph Musimbaggo**	Factors Associated with Blood Pressure Control in Patients with Hypertension and HIV at a Large Urban HIV Clinic in Uganda. Link to article (3)	Manan Pareek,^1^Dinesh Neupane,^1^ Michael H. Olsen^1^

**Swagata Kumar Sahoo**	Financial Implications of Protocol-based Hypertension Treatment: An Insight into Medication Costs in Public and Private Health Sectors in India. Link to article (21)	Andrew Moran,^4^ Dagmara Hering^1^

**Editorial**	Emerging Authors Program for Building Cardiovascular Disease Prevention and Management Research Capacity in Low- and Middle-Income Countries: A Collaboration of the U.S. Centers for Disease Control and Prevention, the Lancet Commission on Hypertension Group, Resolve to Save Lives, and the World Hypertension League. Link to article (0)	EAP Collaborators^1,2,3,4,5^

**Isaac Derickk Kimera**	Integrated Multi-Month Dispensing of Antihypertensive and Antiretroviral Therapy to Sustain Hypertension and HIV Control. Link to article (17)	Christian Delles^1^

**Rim Ghammem**	Effectiveness of a 3-year Community-based Intervention for Blood Pressure Reduction among Adults: A Repeated Cross-sectional Study with a Comparison Area. Link to article (8)	Dinesh Neupane^1^

**Azuka S Adeke**	Socio-demographic and Lifestyle Factors Associated with Hypertension in Nigeria: Results from a Country-wide Survey.Link to article (48)	Dinesh Neupane^1^

**Sarra Soua**	The Prevalence of High Blood Pressure and its Determinants among Tunisian Adolescents. Link to article (19)	Fernando Martinez Garcia^1^

**Ruqayya Nasir Sani**	Rural-urban Difference in the Prevalence of Hypertension in West Africa: A Systematic Review and Meta-analysis. Link to article (51)	Christian Delles^1^

**Editorial**	Emerging Authors Program for Global Cardiovascular Disease Research- A collaboration of the U.S. Centers for Disease Control and Prevention, the Lancet Commission on Hypertension Group, Resolve to Save Lives, and the World Hypertension League. Link to article (3)	EAP Collaborators^1,2,3,4,5^

**Wave I**		

**Sagri Negi**	Prices of Combination Medicines and Single-molecule Antihypertensive Medicines in India’s Private Health Care Sector. Link to article (16)	Dinesh Neupane,^1^ Andrew E. Moran^4^

**Mandar Kannure**	Phone Calls for Improving Blood Pressure Control among Hypertensive Patients Attending Private Medical Practitioners in India: Findings from Mumbai Hypertension Project. Link to article (12)	Angelo Scuteri,^1^ Dinesh Neupane^1^

**Bidisha Das**	Factors Affecting Non-adherence to Medical Appointments among Patients with Hypertension at Public Health Facilities in Punjab, India. Link to article (48)	Dinesh Neupane^1^

**Jhilam Mitra and Abishek Kunwar**	India Hypertension Control Initiative-Hypertension Treatment and Blood Pressure Control in a Cohort in 24 Sentinel Site Clinics. Link to article (67)	Michael H. Olsen^1^

**Chaisiri Angkurawaranon**	Clinical Audit of Adherence to Hypertension Treatment Guideline and Control Rates in Hospitals of Different Sizes in Thailand. Link to article (10)	James Sharman,^1^ Michael H. Olsen^1^

**Worawon Chailimpamontree**	Estimated Dietary Sodium Intake in Thailand: A Nationwide Population Survey with 24-hour Urine Collections. Link to article (52)	Andrew E. Moran^4^

**Gloria P. Giraldo**	Mapping Stages, Barriers and Facilitators to the Implementation of HEARTS in the Americas Initiative in 12 Countries: A Qualitative Study. Link to article (48)	Patricio Lopez-Jaramillo,^1^Michael H. Olsen^1^

**Editorial**	Building Research Capacity within Cardiovascular Disease Prevention and Management in Low- and Middle-income Countries: A collaboration of the US Centers for Disease Control and Prevention, the Lancet Commission on Hypertension Group, Resolve to Save Lives, and the World Hypertension League. Link to article (5)	EAP Collaborators^1,2,3,4,5^

**Juntima Photi**	Evolution of Trans-fatty Acid Consumption in Thailand and Strategies for its Reduction. Link to article (12)	Fernando Martinez Garcia^1^

**Omer M. Tarar**	Understanding the Complexities of Prevalence of Trans Fat and its Control in Food Supply in Pakistan. Link to article (21)	Christian Delles^1^

**Bianca Loge**	Trans Fatty Acid Elimination Policy in Member States of the Eurasian Economic Union: Implementation Challenges and Capacity for Enforcement. Link to article (19)	Christian Delles^1^

**Ashish Krishna**	A Perspective of Private Health Care Providers in the State of Madhya Pradesh on Adopting Key Strategies of the India Hypertension Control Initiative.Link to article (10)	Dagmara Hering^1^

**Khanuengnij Yueayai**	Hospital-based Intervention to Enhance Hypertension Diagnosis in Kalasin Hospital, Thailand, 2017–2019: A pre-post Pilot Intervention Study. Link to article (3)	Andrew E. Moran^4^

**Ramon Martinez**	The Slowdown in the Reduction Rate of Premature Mortality from Cardiovascular Diseases Puts the Americas at Risk of Achieving SDG 3.4: A Population Trend Analysis of 37 Countries from 1990 to 2017. Link to article (71)	Patricio Lopez-Jaramillo^1^

**Editorial**	Global Cardiovascular Disease Prevention and Management: A Collaboration of Key Organizations, Groups, and Investigators in Low- and Middle-income Countries. Link to article (6)	EAP Collaborators^1,2,3,4,5^


*Reflects the number of citations on Google Scholar (Accessed Nov 22, 2025). ^1^Lancet Commission on Hypertension Group; ^2^US Centers for Disease Control and Prevention; ^3^CDC Foundation; ^4^Resolve to Save Lives; ^5^World Hypertension League.

### Best practices

Accessible resources, better communication, and coordination led to a practical, results-driven model guided by best practices.

- **Dedicated Mentors:** Dedicated mentors were key to the program’s success, providing technical expertise, feedback, and encouragement that improved manuscripts and motivated authors. Global mentors added credibility, while local mentors contributed contextual knowledge. Despite logistical challenges, their collaboration was highly valued, demonstrating that engaging both groups can build sustainable publication capacity.
- **Motivated Authors:** Motivated authors with recent projects were vital to success; their focus, openness to feedback, persistence, and collaboration fostered steady progress and long-term career growth.
- **Accessible Resources:** Free, self-paced courses and webinars offered flexible, high-quality learning opportunities. These tools benefited not only EAP participants but also a wider global audience.
- **Coordination:** Effective matching of mentors to mentees and continuous communication by CDC staff ensured smooth implementation.
- **Technical Support:** In Waves I–II, senior reviewers assessed manuscripts for submission readiness; in Wave III, CDC provided checklists, templates, reviewed manuscripts before submission, and helped with responses to peer reviewers.

### Challenges and solutions

- **COVID-19 Pandemic:** The pandemic disrupted data collection, program implementation, and authors’ time. Many Wave I authors were deployed to the COVID-19 response and faced increased workloads. The mentors continued supporting participants despite working long hours at the hospitals; the program extended timelines and adjusted expectations.
- **Language Barriers:** The dominance of English in academic publishing posed a barrier for many authors for whom English was not their first language. To address this challenge, editorial assistance was provided, and the program emphasized clarity, structure, and plain language in the training tools and webinars.
- **Financial Barriers:** High APCs were addressed by identifying free-access journals and funding charges when necessary.
- **Institutional and Infrastructural Limitations:** Challenges such as a lack of institutional support, limited internet and scientific literature access, and time constraints were mitigated through flexible timelines, provision of necessary scientific literature, and using multiple communication platforms when necessary.
- **Mentor-Mentee Challenges:** This collaboration posed communication challenges due to time zone differences and added layers of reviewers. Some authors felt discouraged by the extensive internal review process and the volume of comments/feedback they needed to address. The significant time investment required to revise manuscripts was also noted as burdensome. Mentors and program staff responded by clarifying the purpose of internal review, prioritizing essential revisions, and acknowledging the substantial time investment required from both mentors and mentees. Mentees appreciated the guidance they received, flexibility in scheduling, timely feedback, and a strong support system (8).

## Conclusions

EAP demonstrates that mentorship, virtual training, and strong coordination can build scientific writing and publication capacity in LMICs. With 31 publications in three waves, it effectively bridged the gap between CVD burden and research output. EAP participants are well-positioned to champion future expansion of this model at the local level. By engaging early-career researchers with limited or no prior experience in scientific publishing, they can help build sustainable research capacity and strengthen the ability of LMIC investigators to contribute meaningfully to the global evidence base. Expanding this model locally and regionally, supported by strong collaboration and cross-country networking among EAP authors, can lay the groundwork to enhance its scalability and long-term impact. Consideration of potential funding pathways and alternative support models, such as local mentorship networks, cost-sharing approaches, or institutional partnerships, may also strengthen the feasibility of broader implementation. Free, self-paced tools, such as the Scientific Manuscript Writing course and webinars, remain valuable resources to support broader uptake of these approaches. EAP offers a sustainable, practical framework to advance global health equity in scientific publishing.
